# Endocan as an early biomarker of severity in patients with acute respiratory distress syndrome

**DOI:** 10.1186/s13613-017-0311-4

**Published:** 2017-09-07

**Authors:** Diego Orbegozo, Lokmane Rahmania, Marian Irazabal, Manuel Mendoza, Filippo Annoni, Daniel De Backer, Jacques Creteur, Jean-Louis Vincent

**Affiliations:** Department of Intensive Care, Erasme University Hospital, Université Libre de Bruxelles, Route de Lennik 808, 1070 Brussels, Belgium

**Keywords:** Proteoglycan, Glycocalyx, Pulmonary vasculature, Risk stratification, Acute respiratory failure, Multiple organ failure

## Abstract

**Background:**

Plasma concentrations of endocan, a proteoglycan preferentially expressed in the pulmonary vasculature, may represent a biomarker of lung (dys)function. We sought to determine whether the measurement of plasma endocan levels early in the course of acute respiratory distress syndrome (ARDS) could help predict risk of death or of prolonged ventilation.

**Methods:**

All patients present in the department of intensive care during a 150-day period were screened for ARDS (using the Berlin definition). Endocan concentrations were measured at the moment of ARDS diagnosis (T0) and the following morning (T1). We compared data from survivors and non-survivors and data from survivors with less than 10 days of ventilator support (good evolution) and those who died or needed more than 10 days of mechanical ventilation (poor evolution). Results are presented as numbers (percentages), mean ± standard deviation or medians (percentile 25–75).

**Results:**

Ninety-six consecutive patients were included [median APACHE II score of 21 (17–27) and SOFA score of 9 (6–12), PaO_2_/FiO_2_ ratio 155 (113–206)]; 64 (67%) had sepsis and 51 (53%) were receiving norepinephrine. Non-survivors were older (66 ± 15 vs. 59 ± 18 years, *p* = 0.045) and had higher APACHE II scores [27 (22–30) vs. 20 (15–24), *p* < 0.001] and blood lactate concentrations at study inclusion [2.1 (1.3–4.0) vs. 1.5 (0.9–2.6) mmol/L, *p* = 0.024] than survivors, but PaO_2_/FiO_2_ ratios [150 (116–207) vs. 158 (110–206), *p* = 0.95] were similar in the two groups. Endocan concentrations on the day after ARDS diagnosis were significantly higher in patients with poor evolution than in those with good evolution [12.0 (6.8–18.6) vs. 7.2 (5.4–12.5), *p* < 0.01].

**Conclusion:**

Blood endocan concentrations early in the evolution of ARDS may be a useful marker of disease severity.

**Electronic supplementary material:**

The online version of this article (doi:10.1186/s13613-017-0311-4) contains supplementary material, which is available to authorized users.

## Background

Acute respiratory distress syndrome (ARDS) remains a major concern, with mortality rates around 30–45% [1, 2]. The severity of disease is often assessed using the PaO_2_/FiO_2_ ratio (mainly to optimize treatment strategies), even though the prognostic power of this variable remains low to moderate, with an area under the receiver operating characteristic curve (AUC) of only 0.58 (95% CI 0.56–0.59) in a recent large study [3]. New, early biomarkers of the severity of ARDS are needed, because early optimization of treatment in patients at greatest risk of a poor outcome could improve survival.

ARDS is characterized by important functional and morphological alterations in the pulmonary endothelium that are directly related to mortality [4–8]. Endocan [previously called endothelial cell-specific molecule 1 (ESM-1)] is a proteoglycan that is mainly expressed in the pulmonary microcirculation, where it plays an important role in endothelial homeostasis [9, 10], able to modulate cell adhesion, endothelial permeability and leukocyte migration from the circulation into the tissues [11, 12]. The preferential expression of endocan in the lung microvasculature and repeated observations suggesting the presence of endothelial dysfunction and upregulation of different inflammatory pathways in the pathogenesis of ARDS [7, 13, 14] support a possible role for endocan in the pathophysiology of ARDS.

During experimental endotoxemia, plasma endocan concentrations increase together with concentrations of inflammatory cytokines and concentrations are correlated with the degree of endothelial dysfunction [15]. Moreover, specific endocan blockade with neutralizing antibodies improved survival in a mouse model of peritonitis [16]. Human studies have indicated that plasma endocan concentrations are increased in patients with sepsis and are correlated with the degree of organ dysfunction and mortality [17, 18]. There are few data on endocan concentrations in patients with ARDS. In one prospective cohort of 42 patients with ARDS, endocan concentrations (1 day after the diagnosis of ARDS) were higher in non-survivors than in survivors [19].

We hypothesized that plasma endocan concentrations would be increased in ARDS patients with poor outcomes and could represent a potential early biomarker of ARDS severity.

## Methods

This study was conducted in our 35-bed Department of Intensive Care, which has more than 3200 admissions per year. The Institutional Ethics Committee approved the study (protocol number 2013/269), and written informed consent was obtained from the patient or the patient’s representative or next of kin.

During a 150-day period, a medical team not involved in patient care prospectively screened on a daily basis all patients with an ICU stay of more than 24 h. All adult (>18 years) patients with ARDS as defined by the Berlin definition [3] [PaO_2_/FiO_2_ ratio <300 while receiving mechanical ventilation with a positive end-expiratory pressure (PEEP) of at least 5 cmH_2_O, who had a known risk factor for ARDS and bilateral infiltrates on the chest X ray] were included in the study. If a patient was admitted several times to the ICU during the screening period, only the first admission was considered.

### Endocan concentrations

When a patient met the criteria for ARDS, the closest residual blood sample taken on the same day was obtained from the central hospital laboratory (T0). A second residual blood sample taken at 8 am the next morning (T1) was also obtained. Retrieved tubes were immediately centrifuged and plasma separated and frozen at −80 °C degrees for future analysis. Endocan concentrations were measured using a human specific quantitative sandwich enzyme-linked immunosorbent assay (ELISA) technique (Lunginnov, Lille, France). All measurements were performed in the central immunochemistry laboratory of the hospital. Data provided from Lunginnov (Lille, France) have shown that concentrations of endocan remain stable over time in EDTA tubes stored for less than 72 h at room temperature and support repeated freeze thaw cycles; use of citrate, heparin or plasma tubes leads to underestimation of endocan concentrations.

### Demographic, hemodynamic and clinical data

We collected data on demographics, diagnosis, comorbidities and the presence of infection. All available hemodynamic and respiratory data from patient monitoring systems, including respiratory rate, mean arterial pressure, heart rate, PEEP, and FiO_2_, were recorded at the two study time points (T0 and T1); laboratory data from the same time points were also noted. ARDS was defined as mild when the PaO_2_/FiO_2_ ratio was between 201 and 300, moderate when it was between 101 and 200 and severe when it was ≤100 [3]. The APACHE II score [20] was calculated using the worst data during the first 24 h in the ICU, and the SOFA score [21] was calculated at T0 and T1.

### Outcome measures

We recorded the duration of mechanical ventilation until definitive weaning (able to breath spontaneously for more than 72 h) from respiratory support (including non-invasive ventilation). We also recorded the length of ICU stay and the survival status at the end of the ICU stay. We compared endocan concentrations in ICU survivors and non-survivors but also used a composite outcome measure of mortality and prolonged mechanical ventilation (analogous to the concept of ventilator-free days): survivors who needed <10 days of mechanical ventilation (arbitrarily selected) were classified as having a good evolution and non-survivors or survivors who required >10 days of mechanical ventilation were classified as having a poor evolution.

We also compared plasma endocan concentrations in subgroups of septic and non-septic patients, and trauma and non-trauma patients. Because of the known preferential expression of endocan in the lungs, we evaluated the correlation between endocan plasma concentrations and the PaO_2_/FiO_2_ as an index of lung function at T0. We also explored the role of possible confounding factors, including the degree of organ dysfunction (SOFA score ≥10 vs. <10 [cut-off selected because the median value for the whole population was 9]), the presence of renal failure (SOFA renal score of 0 vs. 1–4), the presence of comorbidities (patients with and without chronic lung disease, comorbid diabetes, hypertension and cardiovascular disease) and the etiology of ARDS [pulmonary (primary) vs. extra-pulmonary (secondary)].

### Statistical analysis

Continuous variables were explored for normality of distribution by looking at the Q-Q plots and using Skewness and Kurtosis tests. Values are presented as means ± standard deviations when normality was confirmed and as medians with percentiles (25–75%) when the distribution was not normal. Categorical data are presented as numbers of events and percentages. Comparisons between groups were performed using *t* tests or Mann–Whitney *U* tests as appropriate. Proportions were compared using a Chi-square test or Fisher’s exact test as appropriate. Correlations between different variables were examined using the Pearson coefficient (*r*). We plotted sensitivity and specificity on a ROC graph and the area under the curve (AUC) was calculated for the different variables to predict mortality or poor evolution. A post hoc analysis was performed to determine cut-off values with high specificity and/or sensitivity for predicting poor outcome or mortality by exploring the data at different points on the ROC curves. A post hoc binary logistic regression (univariate and multivariate analysis) was performed to identify the role of T1 endocan and other variables to predict poor evolution, calculating the odds ratio (OR) and its respective 95% confidence interval (CI). A two-sided *p* value less than 0.05 was considered as significant for all analyses. Statistical analysis was performed using SPSS 22.0 (IBM, New York, NY) software.

## Results

**Fig. 1:**
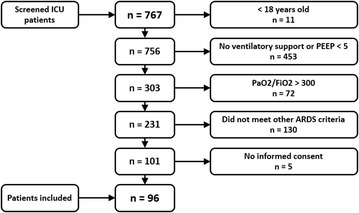
Screening flowchart. PEEP: positive end-expiratory pressure

Ninety-six patients met ARDS criteria during the screening period and were included in the study (Fig. 1). The median time between ICU admission and inclusion in the study (confirmed diagnosis of ARDS = T0) was 3 (0–12) h. The median time between T0 and T1 samples was 24 (16–24) h.

The main baseline characteristics of the patients at T0 are shown in Table 1. Non-survivors were older (66 ± 15 vs. 59 ± 18 years, *p* = 0.045) and had higher APACHE II scores [27 (22–30) vs. 20 (15–24), *p* < 0.001] and blood lactate concentrations [2.1 (1.3–4.0) vs. 1.5 (0.9–2.6), *p* = 0.024] than survivors but similar PaO_2_/FiO_2_ ratios [150 (116–207) vs. 158 (110–206), *p* = 0.95].

**Table 1:** Main characteristics (at time of inclusion) and outcomes of the patients with ARDS

	Total *n* = 96	Survivors *n* = 64	Non-survivors *n* = 32	*p*	Good evolution *n* = 54	Poor evolution *n* = 42	*p*
Age (years)	61 ± 17	59 ± 18	66 ± 15	*0.045*	59 ± 18	64 ± 15	0.219
Male *n* (%)	64 (67)	44 (69)	20 (63)	0.647	39 (72)	25 (60)	0.275
Trauma *n* (%)	8 (8)	7 (11)	1 (3)	0.262	5 (9)	3 (7)	1.000
Primary ARDS *n* (%)	45 (47)	31 (48)	14 (44)	0.828	25 (46)	20 (48)	1.000
Sepsis *n* (%)	64 (67)	39 (61)	25 (78)	0.111	33 (61)	31 (74)	0.275
Chronic lung disease *n* (%)	16 (17)	7 (11)	9 (28)	*0.044*	6 (11)	10 (24)	0.166
Chronic kidney disease *n* (%)	15 (16)	9 (14)	6 (19)	0.767	7 (13)	8 (19)	0.572
Active cancer *n* (%)	4 (4)	4 (6)	0 (0)	0.298	3 (6)	1 (2)	0.629
APACHE II score	21 (17–27)	20 (15–24)	27 (22–30)	*<0.001*	20 (15–22)	27 (22–31)	*<0.001*
SOFA score	9 (6–12)	8 (5–11)	9 (7–13)	0.070	8 (4–10)	10 (7–13)	*0.004*
Respiratory rate (bpm)	24 ± 7	24 ± 7	23 ± 6	0.773	24 ± 7	24 ± 6	0.992
FiO_2_ (%)	50 (40–60)	50 (50–60)	50 (40–61)	0.913	50 (50–60)	50 (40–60)	0.794
PEEP (cmH_2_0)	8 (5–8)	8 (5–8)	6 (5–10)	0.264	8 (5–8)	6 (5–10)	0.574
Arterial pH	7.38 ± 0.09	7.40 ± 0.08	7.33 ± 0.10	0.002	7.40 ± 0.07	7.35 ± 0.11	0.009
PaO_2_ (mmHg)	79 (67–98)	79 (65–97)	79 (68–107)	0.724	80 (65–98)	77 (68–97)	0.631
PaCO_2_ (mmHg)	38 (35–44)	39 (36–44)	38 (32–45)	0.508	40 (36–45)	38 (34–44)	0.252
PaO_2_/FiO_2_ ratio	155 (113–206)	158 (110–206)	150 (116–207)	0.946	164 (108–214)	143 (115–198)	0.413
ARDS				0.870			0.600
Severe ARDS *n* (%)	15 (16)	11 (17)	4 (12)		9 (17)	6 (14)	
Moderate ARDS *n* (%)	56 (58)	36 (56)	20 (63)		29 (54)	27 (64)	
Mild ARDS *n* (%)	25 (26)	17 (27)	8 (25)		16 (29)	9 (22)	
MAP (mmHg)	81 ± 13	80 ± 13	83 ± 13	0.379	81 ± 13	82 ± 14	0.645
Heart rate (bpm)	94 ± 21	95 ± 21	92 ± 21	0.456	93 ± 21	96 ± 21	0.520
CVP (mmHg)	10 (8–13)	10 (8–14)	10 (8–12)	0.678	10 (8–13)	10 (9–13)	0.683
Norepinephrine, *n* (%)	51 (53)	35 (55)	16 (50)	0.828	28 (52)	23 (55)	0.838
Norepinephrine (mcg/Kg/min)	0.02 (0.00–0.22)	0.02 (0.00–0.17)	0.02 (0.00–0.35)	0.676	0.01 (0.00–0.18)	0.07 (0.00–0.30)	0.391
Lactate (mmol/L)	1.7 (1.1–3.0)	1.5 (0.9–2.6)	2.1 (1.3–4.0)	*0.024*	1.3 (0.9–2.7)	2.1 (1.2–3.3)	*0.028*
Creatinine (mg/dL)	1.0 (0.7–1.4)	0.9 (0.7–1.3)	1.2 (0.8–1.7)	0.213	0.9 (0.7–1.2)	1.2 (0.7–1.7)	0.070
Renal failure *n* (%)	40 (42)	23 (37)	17 (53)	0.131	16 (30)	24 (57)	*0.012*
Total bilirubin (mg/dL)	0.7 (0.5–1.7)	0.6 (0.5–1.4)	1.1 (0.6–1.9)	*0.037*	0.6 (0.5–1.0)	1.1 (0.5–1.9)	0.096
Platelets (x10^3^/µL)	168 (105–225)	182 (107–280)	136 (80–179)	*0.011*	183 (107–280)	136 (93–183)	*0.040*
Leukocytes (cells x10^3^/µL)	12.0 (8.5–15.4)	11.2 (7.7–15.3)	12.5 (8.9–16.1)	0.455	11.2 (8.2–15.2)	12.4 (8.7–15.5)	0.624
Duration of mechanical ventilation (days)	4.5 (2.4–8.2)	4.3 (2.5–7.2)	5.6 (2.2–8.6)	0.756	3.6 (1.8–6.2)	7.2 (2.7–16.9)	*<0.001*
ICU length of stay (days)	6.4 (4.4–11.0)	7.1 (4.6–12.4)	6.0 (2.6–9.6)	0.140	6.0 (4.4–8.9)	8.3 (4.1–17.4)	0.109

*n* number of patients, *ARDS* acute respiratory distress syndrome, *FiO*_*2*_ inspired oxygen fraction, *PEEP* positive end-expiratory pressure, *PaO*_*2*_ arterial oxygen pressure, *PaCO*_*2*_ arterial carbon dioxide pressure, *MAP* mean arterial pressure, *CVP* central venous pressure, *ICU* intensive care unit

Statistically significant *p* values (<0.05) are shown in italics

**Fig. 2:**
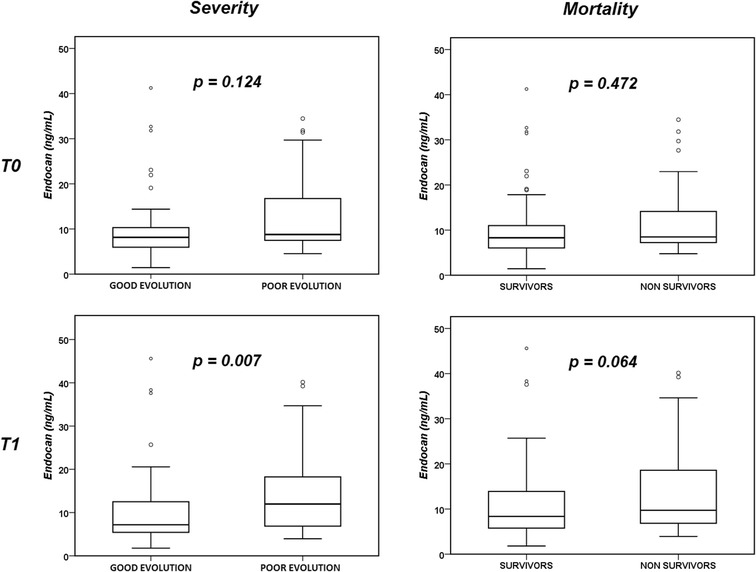
Comparison of endocan concentrations at T0 and T1 in survivors and non-survivors and in patients with poor and good evolution

**Fig. 3:**
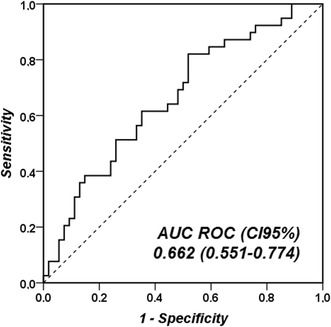
Receiver operating characteristic (ROC) curve for T1 endocan concentrations to predict poor evolution

Plasma endocan concentrations were statistically significantly higher in patients with poor evolution than in those with good evolution at T1, but not at T0 (Fig. 2). This pattern was similar, but differences were not statistically significant, in the comparison of endocan concentrations in non-survivors and survivors. The change in endocan concentrations between T0 and T1 was not significantly different in patients with poor and good evolution (*p* = 0.127) or in non-survivors and survivors (*p* = 0.476). The AUC of T1 endocan concentrations for prediction of poor evolution is shown in Fig. 3. The AUCs for the APACHE II score, the T0 SOFA score and the T0 PaO_2_/FiO_2_ ratio are shown in Additional file 1: Table S1. In the multivariate analysis, APACHE II score (but not endocan at T1) was retained as the best predictor of poor evolution (OR 1.149 [1.067–1.237], *p* < 0.01). A cut-off value of endocan of 6 ng/mL at T0 and T1 had good sensitivity to exclude poor outcome or death, and a cut-off value of 14 ng/mL at T0 and T1 had good specificity for poor outcome or death, with acceptable negative predictive values at T1, but modest positive predictive values (Additional file 1: Table S2).

**Fig. 4:**
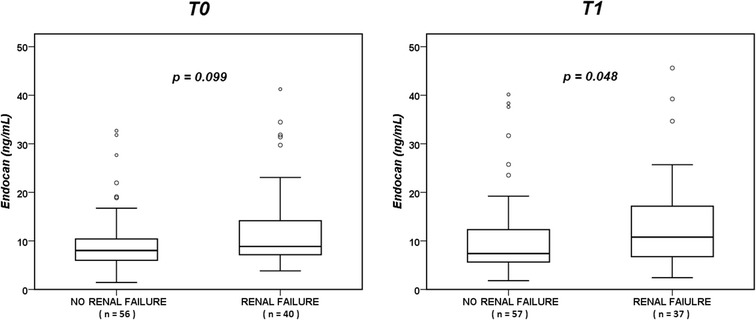
Endocan concentrations at T0 and T1 in patients with (renal SOFA subscore 1–4) and without (renal SOFA subscore 0) renal failure

Endocan concentrations at T0 and T1 were higher in patients with higher SOFA scores than in those with lower SOFA scores (using a cut-off of 10 points) (Additional file 1: Fig. S1). At T0, they were also higher in patients with sepsis than in those without, but were no different in trauma and non-trauma patients, patients with or without chronic lung comorbidities, patients with primary versus secondary ARDS (Additional file 1: Fig S2), patients with or without diabetes (*p* = 0.198), patients with or without arterial hypertension (*p* = 0.407), and patients with or without coronary heart disease (*p* = 0.473). At T1, patients with higher renal SOFA scores had higher endocan concentrations than did patients with a SOFA renal score of 0 (Fig. 4).

**Fig. 5:**
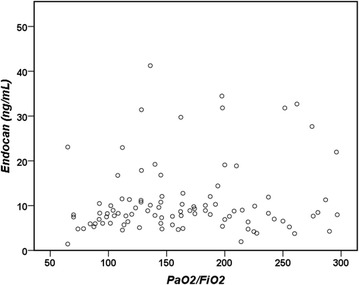
Correlation of endocan concentrations with PaO_2_/FiO_2_ ratio at T0 (*r* = 0.137, *p* = 0.18)

At T0, there was a modest correlation between endocan concentrations and the renal (*r* = 0.235, *p* = 0.001) and the total (*r* = 0.332, *p* < 0.001) SOFA scores. Endocan concentrations at T0 were not correlated with the PaO_2_/FiO_2_ ratio at T0 (Fig. 5) (*r* = 0.137, *p* = 0.18), with the PaO_2_/FiO_2_ ratio at T1 (*r* = 0.191, *p* = 0.07), or with the change in the PaO_2_/FiO_2_ ratio between T0 and T1 (*r* = 0.095, *p* = 0.36). The changes in endocan concentrations between T0 and T1 were not significantly correlated with the changes in PaO_2_/FiO_2_ ratios between T0 and T1 (*r* = 0.126, *p* = 0.23).

In the subgroups of septic and non-septic patients (*n* = 64 and *n* = 32, respectively), there were no significant differences in endocan concentrations at T0 or T1 in patients with poor evolution or good evolution (Additional file 1: Table S3). In the subgroup of non-trauma patients (*n* = 88), endocan concentrations at T1 were higher in patients with a poor evolution than in those with a good evolution (*p* = 0.03), but not at T0 (Additional file 1: Table S3).

## Discussion

Our results demonstrate that plasma endocan concentrations measured early in the course of ARDS can reflect disease severity and predict poor evolution, as assessed by death or prolonged dependence on mechanical ventilation.

Data on endocan levels in patients with ARDS are limited. A retrospective study of 24 trauma patients reported that patients who developed ARDS had lower endocan levels on admission than matched patients without ARDS [22]. A prospective study in 20 septic ICU patients reported lower endocan concentrations at admission in patients who had developed respiratory failure by day 3 after admission than in those who had not; the endocan level at admission was correlated with the decrease in PaO_2_/FiO_2_ ratio on days 2 and 3 [11]. These studies only measured endocan concentrations on admission. In a more recent study in patients with sepsis, endocan concentrations were measured on admission and after 72 h or on development of new organ dysfunction; an increase in endocan concentrations was associated with development of ARDS [23]. Our results show that endocan concentrations are higher in patients with more severe ARDS who require prolonged mechanical ventilation. Similarly, in a small prospective cohort of 42 patients with ARDS (mainly due to sepsis), endocan concentrations (1 day after the diagnosis of ARDS) were higher in non-survivors than in survivors [19].

We measured endocan concentrations at two points during the early phase of ARDS, a period when prognostication is difficult. Several studies have shown that the PaO_2_/FiO_2_ ratio has only mild to moderate power to predict bad outcomes [3, 24, 25]. A score that combines PaO_2_/FiO_2_ ratios, cardiovascular dysfunction and age has been proposed, but data need to be collected for 3 days following diagnosis [26]. Others have suggested combining different clinical variables at the moment of ARDS diagnosis [25], but external validation is missing. Our data show that endocan concentrations have prognostic power already on the first day after ARDS diagnosis and that there was no correlation of endocan concentrations with PaO_2_/FiO_2_ ratios on admission or with changes in the ratio over the first 2 days after diagnosis.

Our data confirm previous findings of high endocan concentrations in septic patients. In an early study, Scherpereel and colleagues [17] showed that human in vitro endothelial cells secreted endocan after stimulation by lipopolysaccharide and tumor necrosis factor-alpha. They also reported higher circulating endocan concentrations in septic patients than in healthy donors or patients with isolated systemic inflammatory response syndrome (SIRS) [17]. Mihajlovic and colleagues reported that higher initial endocan concentrations were related to later development of organ dysfunction and mortality in patients with sepsis [18]. However, although T0 endocan concentrations were higher in patients with sepsis than in those without, there were no differences in endocan concentrations at T0 and T1 in patients with poor or good evolution in the sepsis subgroup.

We observed higher endocan concentrations in patients with than in those without renal failure. In healthy human tissues, endocan is preferentially expressed in the lung endothelium, but also in the glomerular endothelial and tubular epithelial cells in the kidneys [9, 10]. A recent study showed that in patients who had received a kidney transplant, endocan concentrations were directly correlated with more advanced stages of chronic kidney disease [27]. In pathological conditions, renal expression of endocan may potentially be increased or renal excretion reduced, but there are no data in this field. Based on currently available data, renal function should be taken into consideration when interpreting endocan concentrations.

In addition to its potential role in the pathogenesis of ARDS and other inflammatory conditions, endocan may also be involved in the pathogenesis of other conditions. Recent data suggest that endocan may have a role in chronic cardiovascular disease [12], hypertension or diabetes, but endocan levels were similar in patients with and without these comorbid conditions in our database. One human study has suggested that endocan is also expressed in highly proliferative tissues, such as the neo-vasculature or the lymph nodes [10]. It has been shown that in some neoplasms, endocan is preferentially expressed in the tumor endothelium and its expression is regulated by tumor-derived factors [28]. Exposure of mice to high concentrations of endocan can also induce the development of tumors [29]. This observation may explain why elevated plasma concentrations have been found in patients with different types of cancer and are associated with the probability of survival [30–32]. The prevalence of active cancer in our population was low and endocan concentrations were no different in these patients compared to those without cancer (data not shown); nevertheless, this factor may be relevant in oncologic ICUs.

Our study has several limitations. First, ARDS is a heterogeneous syndrome including various pathogenic mechanisms that may or may not be related to the endocan pathway. Second, we did not measure endocan concentrations after 24 h and therefore have no information on concentrations at later time points, but we were focusing on endocan as an early biomarker, because patient outcomes are highly influenced by subsequent clinical evolution. Third, including a control group of non-ARDS patients may have provided interesting information regarding the role of endocan as a diagnostic biomarker, but our aim was to determine whether endocan could be used as an early biomarker of severity in patients already diagnosed with ARDS. Finally, our study may have lacked power to detect some differences in the early stages of ARDS and in the analyzed subgroups, as suggested by the tendency for endocan concentrations to be higher in patients with worse outcomes even at the time of ARDS diagnosis.

## Conclusion

In patients with ARDS, plasma endocan concentrations 24 h after diagnosis may be useful to predict poor evolution.

## Additional file

**Additional file 1.** Tables and figures showing AUCs for different variables; sensitivity, specificity, PPV and NPV for different endocan cut-off values; endocan levels in pre-defined sub-groups; and endocan concentrations at T0 and T1 according to the presence of organ dysfunction.

## Abbreviations

APACHE: acute physiology and chronic health evaluation
ARDS: acute respiratory distress syndrome
AUC: area under the curve
CI: confidence interval
CVP: central venous pressure
EDTA: ethylenediaminetetraacetic acid
ELISA: enzyme-linked immunosorbent assay
ESM-1: endothelial cell-specific molecule 1
FiO_2_: inspired oxygen fraction
ICU: intensive care unit
MAP: mean arterial pressure
PaO_2_: arterial oxygen pressure
PaCO_2_: arterial carbon dioxide pressure
PEEP: positive end-expiratory pressure
ROC: receiver operating characteristic
SIRS: systemic inflammatory response syndrome
SOFA: sequential organ failure assessment
