# Supplementary material for: Endocan as an early biomarker of severity in patients with acute respiratory distress syndrome
Source: Ann Intensive Care. 2017 Sep 7;7:93. doi: 10.1186/s13613-017-0311-4 (PMC5589715; doi:10.1186/s13613-017-0311-4)
Supplement: Supplementary file 1 — Additional file 1. Tables and figures showing AUCs for different variables; sensitivity, specificity, PPV and NPV for different endocan cut-off values; endocan levels in pre-defined sub-groups; and endocan concentrations at T0 and T1 according to the presence of organ dysfunction. [file 13613_2017_311_MOESM1_ESM.docx]

**ENDOCAN AS AN EARLY BIOMARKER OF SEVERITY IN PATIENTS WITH**

**ACUTE RESPIRATORY DISTRESS SYNDROME (ARDS)**

**Orbegozo et al**

***Electronic supplemental material (ESM)***

**Table S1.** Areas under the receiver operating characteristics curves (AUC ROC) for different variables to predict mortality or poor evolution (death or > 10 days mechanical ventilation)

| **Variable** | **Mortality** | | **Poor evolution** | |
| --- | --- | --- | --- | --- |
|  | **AUC ROC** | **CI 95%** | **AUC ROC** | **CI 95%** |
| **APACHE II score** | 0.722 | 0.610-0.834 | 0.764 | 0.662-0.865 |
| **SOFA score at T0** | 0.613 | 0.494-0.733 | 0.670 | 0.562-0.779 |
| **PaO_2_/FiO_2_ ratio at T0** | 0.504 | 0.383-0.626 | 0.451 | 0.335-0.567 |
| **Endocan at T0** | 0.545 | 0.424-0.667 | 0.592 | 0.478-0.706 |
| **Endocan at T1** | 0.620 | 0.500-0.740 | 0.662 | 0.551-0.774 |

**Table S2.** Sensitivity, specificity, positive predictive value (PPV) and negative predictive value (NPV) for different endocan cut-off values to predict poor outcome and mortality in patients with ARDS. (Results for the proposed cut-off values of 6 and 14 ng/mL are shown in bold).

| **Study time-point** | **Endocan cut-off value (ng/mL)** | **Number of cases below cut-off value** | **POOR EVOLUTION** | | | | **MORTALITY** | | | |
| --- | --- | --- | --- | --- | --- | --- | --- | --- | --- | --- |
|  |  |  | **Sn (%)** | **Sp (%)** | **PPV (%)** | **NPV (%)** | **Sn (%)** | **Sp (%)** | **PPV (%)** | **NPV (%)** |
| **T0** | 4 | 5 | 100 | 7.4 | 45.7 | 100 | 100 | 6.3 | 34.8 | 100 |
|  | 5 | 14 | 90.5 | 18.5 | 46.3 | 71.4 | 90.6 | 17.2 | 35.4 | 78.6 |
|  | ***6*** | ***21*** | ***83.3*** | ***25.9*** | ***46.7*** | ***66.7*** | ***81.3*** | ***23.4*** | ***34.7*** | ***71.4*** |
|  | 7 | 30 | 78.6 | 38.9 | 50.0 | 70.0 | 75.0 | 34.4 | 36.4 | 73.3 |
|  | 8 | 43 | 61.9 | 50.0 | 49.1 | 62.8 | 56.3 | 45.3 | 34.0 | 67.4 |
|  | 9 | 57 | 42.9 | 61.1 | 46.2 | 57.9 | 37.5 | 57.8 | 30.8 | 64.9 |
|  | 10 | 63 | 40.5 | 70.4 | 51.5 | 60.3 | 37.5 | 67.2 | 36.4 | 68.3 |
|  | 11 | 71 | 35.7 | 81.5 | 60.0 | 62.0 | 31.3 | 76.6 | 40.0 | 69.0 |
|  | 12 | 76 | 26.2 | 83.3 | 55.0 | 59.2 | 25.0 | 81.3 | 40.0 | 68.4 |
|  | ***14*** | ***79*** | ***26.2*** | ***88.9*** | ***64.7*** | ***60.8*** | ***25.0*** | ***85.9*** | ***47.1*** | ***69.6*** |
|  | 16 | 80 | 23.8 | 88.9 | 62.5 | 60.0 | 21.9 | 85.9 | 43.8 | 68.8 |
|  | 20 | 85 | 14.3 | 90.7 | 54.5 | 57.6 | 15.6 | 90.6 | 45.5 | 68.2 |
| **T1** | 4 | 8 | 97.4 | 11.1 | 44.2 | 85.7 | 96.6 | 9.4 | 32.6 | 85.7 |
|  | 5 | 15 | 92.3 | 20.4 | 45.6 | 78.6 | 93.1 | 18.8 | 34.2 | 85.7 |
|  | ***6*** | ***28*** | ***84.6*** | ***40.7*** | ***50.8*** | ***78.6*** | ***82.8*** | ***35.9*** | ***36.9*** | ***82.1*** |
|  | 7 | 37 | 71.8 | 50.0 | 50.9 | 71.1 | 69.0 | 45.3 | 36.4 | 76.3 |
|  | 8 | 41 | 64.1 | 51.9 | 49.0 | 66.7 | 62.1 | 48.4 | 35.3 | 73.8 |
|  | 9 | 49 | 61.5 | 64.8 | 55.8 | 70.0 | 62.1 | 60.9 | 41.9 | 78.0 |
|  | 10 | 55 | 51.3 | 68.5 | 54.1 | 66.1 | 48.3 | 64.1 | 37.8 | 73.2 |
|  | 11 | 56 | 51.3 | 70.4 | 55.6 | 66.7 | 48.3 | 65.6 | 38.9 | 73.7 |
|  | 12 | 59 | 48.7 | 74.1 | 57.6 | 66.7 | 44.8 | 68.8 | 39.4 | 73.3 |
|  | ***14*** | ***67*** | ***38.5*** | ***81.5*** | ***60.0*** | ***64.7*** | ***34.5*** | ***76.6*** | ***40.0*** | ***72.1*** |
|  | 16 | 72 | 33.3 | 87.0 | 65.0 | 64.4 | 31.0 | 82.8 | 45.0 | 72.6 |
|  | 20 | 80 | 20.5 | 92.6 | 66.7 | 61.7 | 20.7 | 90.6 | 50.0 | 71.6 |

**Table S3.** Endocan levels in pre-defined sub-groups of patients according to outcome

|  | **SEPSIS** | | | | | | | |
| --- | --- | --- | --- | --- | --- | --- | --- | --- |
|  | SURVIVOR  n=39 | | NON-SURVIVOR  n=25 | P | GOOD EVOLUTION  n=33 | POOR EVOLUTION  n=31 | P | |
| Endocan at T0 (ng/mL) | 8.8  (6.1-12.8) | | 8.7  (7.7-11.3) | 0.774 | 8.3  (6.0-11.9) | 8.9  (7.7-16.8) | 0.208 | |
| Endocan at T1 (ng/mL) | 9.0  (5.9-16.7) | | 10.8  (6.8-16.6) | 0.486 | 8.8  (5.9-14.0) | 12.0  (7.4-20.0) | 0.094 | |
|  | **NO SEPSIS** | | | | | | | |
|  | SURVIVOR  n=25 | | NON-SURVIVOR  n=7 | P | GOOD EVOLUTION  n=21 | POOR EVOLUTION  n=11 | P | |
| Endocan at T0 (ng/mL) | 8.2  (5.7-9.8) | | 7.5  (5.1-27.7) | 0.656 | 8.0  (5.7-9.8) | 8.2  (5.1-11.5) | 0.584 | |
| Endocan at T1 (ng/mL) | 6.0  (4.3-8.5) | | 7.8  (5.8-19.2) | 0.148 | 5.8  (4.3-8.5) | 7.8  (5.8-18.6) | 0.104 | |
|  | **TRAUMA** | | | | | | | |
|  | SURVIVOR  n=7 | | NON-SURVIVOR  n=1 | P | GOOD EVOLUTION  n=5 | POOR EVOLUTION  n=3 | P | |
| Endocan at T0 (ng/mL) | 9.0  (6.0-9.8) | | 5.2  (5.2-5.2) | 0.500 | 8.5  (6.0-9.0) | 9.3  (5.2-11.4) | 0.571 | |
| Endocan at T1 (ng/mL) | 6.4  (4.7-12.3) | | 6.9  (6.9-6.9) | 1.000 | 5.3  (4.7-6.4) | 12.3  (6.9-14.9) | 0.071 | |
|  | **NO TRAUMA** | | | | | | |  |
|  | SURVIVOR  n=57 | NON-SURVIVOR  n=31 | | P | GOOD EVOLUTION  n=49 | POOR EVOLUTION  n=39 | P |  |
| Endocan at T0 (ng/mL) | 8.2  (6.0-11.1) | 8.7  (7.5-16.7) | | 0.366 | 8.0  (6.0-10.5) | 8.7  (7.5-16.8) | 0.138 |  |
| Endocan at T1 (ng/mL) | 8.4  (5.8-14.0) | 10.8  (6.7-18.9) | | 0.088 | 8.3  (5.8-13.6) | 10.8  (6.8-18.9) | 0.027 |  |

**Figure S1.** Comparison of endocan concentrations at T0 and T1 according to the presence of organ dysfunction. SOFA: sequential organ failure assessment


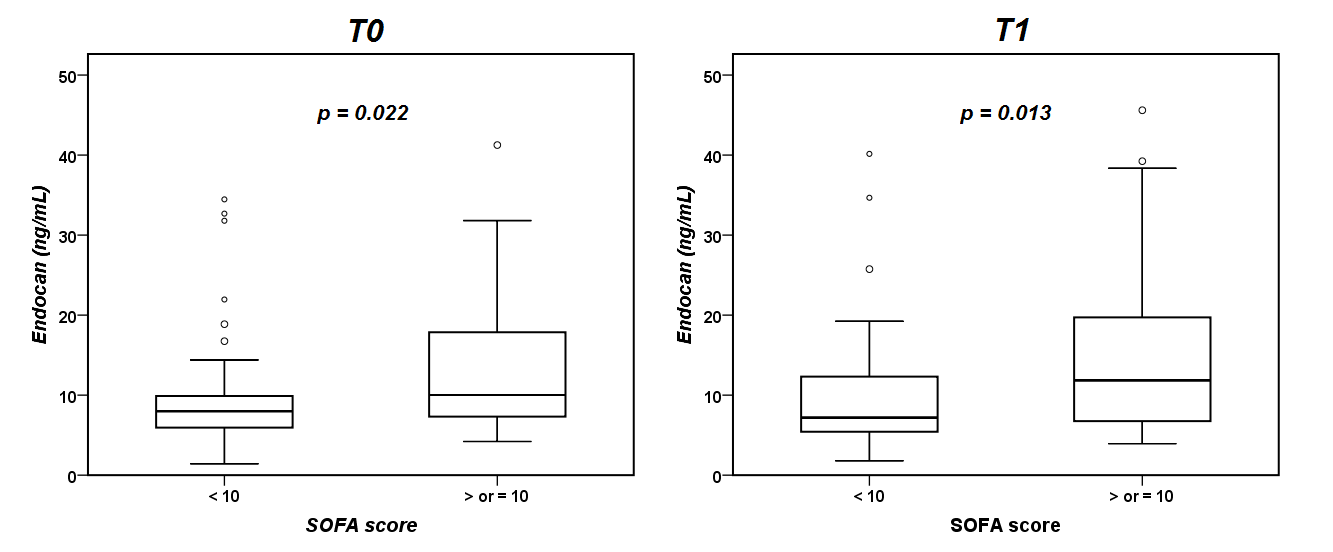


**Figure S2.** Endocan concentrations in different sub-groups of patients at T0

**
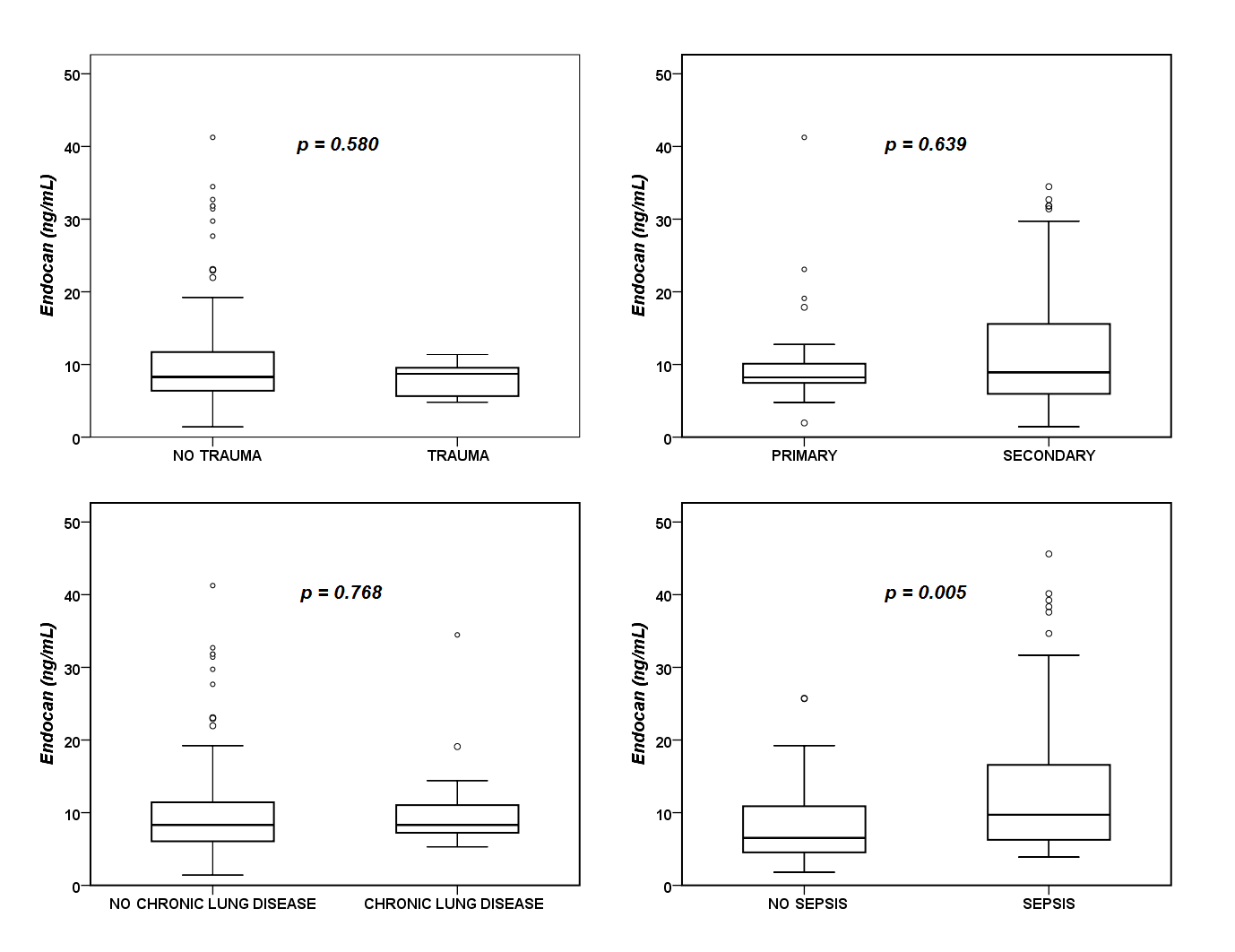
**
